# Prospective study assessing the validity of accelerated 2D Fast Spin Echo (2D FSE) based high-resolution knee MRI and T2 mapping using deep learning reconstruction

**DOI:** 10.1186/s12891-025-09482-2

**Published:** 2026-01-15

**Authors:** Xiaxia Wu, Weiyin Vivian Liu, Kejun Wang, Jiawei Jiang, Changsheng Liu, Yunfei Zha

**Affiliations:** 1 Department of Radiology, Renmin Hospital of Wuhan University, Wuhan, 430060 China; 2 MR Research, GE Healthcare, Beijing, 100176 China; 3 School of Computer Science, Wuhan University, Wuhan , China

**Keywords:** Deep learning reconstruction (DLR), High-resolution, Knee, Cartilage lesions, T2 mapping, Arthroscopy

## Abstract

**Background:**

A routine fast spin-echo (FSE) MRI protocol is widely used to evaluate structural injuries of the knee. While adding a T2 mapping sequence to this protocol increases sensitivity for detecting early cartilage lesions, it is time-consuming. Deep learning reconstruction (DLR) can provide accelerated, high-quality imaging but requires further clinical validation. This study aimed to evaluate the image quality and diagnostic performance of an accelerated, high-resolution 2D FSE protocol using DLR. Additionally, it sought to determine whether a routine MR imaging protocol combining an accelerated T2 mapping sequence with DLR could improve diagnostic performance for the detection of cartilage lesions, using arthroscopy as the reference standard.

**Methods:**

A total of 92 patients underwent 2D FSE based routine knee imaging on 3.0T MRI, 39 of whom also underwent sagittal T2 mapping with different k-space-based acceleration factor of 2 or 3 and then reconstructed using conventional and deep learning reconstruction algorithms as FSE_O_, FSE_DLR_, T2_O − ARC=2,3_, and T2_DLR − ARC=2,3_ and arthroscopy of the knee joint. Two radiologists subjectively and objectively evaluated both FSE_O_ and FSE_DLR_. The inter-reader agreement for each pathology and image quality score was assessed using Cohen’s κ. The objective metrics (SNR/CNR) between sequences was analyzed using paired t-test or Wilcoxon signed-rank test according to data normality. A two-sided p-value of less than 0.05 was considered statistically significant. Additionally, diagnostic performance of routine knee MR images and DLR or non-DLR T2 measurements respectively for grading knee cartilage were also compared. Articular cartilage was categorized according to International Cartilage Repair Society (ICRS). Each articular surface was then evaluated at arthroscopy. Receiver operating characteristic curve (ROC) was used to analyze diagnostic performance using arthroscopic results as reference.

**Results:**

Inter-reader agreement of subjective assessment ranged from 0.70 (95% CI: 0.46–0.94,) to 0.89 (95% CI: 0.79–0.99,) and higher score on FSE_DLR_ than FSE_O_. Sharpness for FSE_DLR_ was rated to be excellent (median Likert score, 5; range, 5–5), higher compared to FSE_O_ (median Likert score, 5; range,4–5),(*P* < 0.001)). Both SNR and CNR of FSE_DLR_ were higher than those of FSE_O_ (*P* < 0.001). Inter-reader agreement was almost perfect, withκvalues between 0.94 (95% CI: 0.85-1.0) to 1.00 (95% CI: 1.0–1.0) for the detection of internal derangement and substantial to almost perfect between 0.70 (95% CI:0.52–0.88) and 0.93 (95% CI:0.85-1.0) for the assessment of cartilage defects. FSE_DLR_ (Reader 1 AUC, 0.77; 95% CI: 0.69–0.84 and Reader 2 AUC, 0.86; 95% CI: 0.78–0.91) had higher diagnostic performance than FSE_O_ (Reader 1 AUC, 0.74; 95% CI: 0.66–0.81 and Reader 2 AUC, 0.80; 95% CI: 0.72–0.86; *P* = 0.005) for articular cartilage lesions. Moreover, Reader 1 achieved the higher diagnostic efficacy (AUC, 0.84; 95% CI: 0.76–0.90) in differentiating normal-appearing from injury-visible cartilage when using both routine FSE_DLR_ images and T2_DLR − ARC=2,_while Reader 2 achieved an AUC of 0.86 (95% CI: 0.78–0.91) with routine FSE_DLR_ images.

**Conclusion:**

Our preliminary results indicate that the accelerated DLR FSE protocol provided diagnostic performance equivalent to the standard protocol for internal derangement, with potential improvement for the detection of cartilage lesions, while delivering higher image quality and quantitative T2 data within a clinically feasible scan time. These findings suggest its potential value for a comprehensive and efficient assessment of knee injury.

**Supplementary Information:**

The online version contains supplementary material available at 10.1186/s12891-025-09482-2.

## Introduction

Knee fast spin echo based magnetic resonance imaging (FSE-MRI) is widely used to evaluate the structural injuries in meniscus, ligament and cartilage due to good tissue contrast and high spatial resolution [1, 2]. A routine knee FSE-based imaging protocol takes approximately 7–10 min on 3.0T MR [1–4]. However, the diagnostic utility of conventional MRI is often compromised by its lengthy acquisition times. As the middle-aged and older patients experience knee pain and mobility loss, their discomfort frequently leads to motion artifacts and an inability to tolerate the complete examination [1, 2, 5, 6]. Morphologic MRI may not detect specific conditions, such as early articular cartilage degeneration and healing conditions after cartilage repair. Early detection helps reverse cartilage injury when in-time intervention [7]. Mechanical wear from joint use brings inevitable and irreversible loss of structures especially for an adult articular cartilage that does not have capacity of self-repaired [8].Quantitative MRI imaging such as T2 mapping has potential to early detect changes in the extracellular matrix of articular cartilage at the the molecular level before the morphological changes, and thus achieves early diagnosis of articular cartilage degeneration [9–11]. The addition of a T2 mapping sequence to a routine MR imaging protocol at 3.0 T can improve the detection of cartilage lesions within the knee joint and may be especially useful in certain patient populations where the identification of early cartilage degeneration is clinically important [10]. However, long acquisition time leads to low patient compliance (motion and displacement during one scan), especially pediatric and aging populations, restricting implementation of in-plane high resolution FSE-based structural and quantitative MRI [12, 13].

To speed up, parallel imaging (PI), compressed sensing (CS) and simultaneous multi-layer acquisition are used to achieve both under-sampling and high-frequency data scarcity using an acceleration factor of 2–3 but possibly contribute to reduced signal-noise ratio (SNR) and increased image blurring [5, 14–16]. In other words, missing anatomical textures of lesions or defects may occur due to widely-used fourier transform as conventional reconstruction (CR) [5, 14, 16]. Recently, deep learning reconstruction (DLR) algorithm via learning regularizer from a set of training data generates better image quality via removal of noise and truncation artifacts in musculoskeletal systems such as knee, shoulder and spine [1, 4, 12, 15, 17–22]. The combination of PI approaches such as GRAPPA and deep neural network can accelerate imaging acquisition without losing diagnostic efficacy compared to two methods alone [23, 24]. In particular, DLR proton weighted imaging (PDWI) and fat-saturated T2-weighted imaging (fs T2WI) acquired with one number of excitation (NEX) has equivalent image quality of CR 4-NEX-acquired PDWI and (fs T2WI) [25].Thus, applications of DLR in both anatomical and quantitative MR images should be cautious in clinical practice despite of advantages like relatively short scan time, elevation of image quality, diagnosis confidence and accuracy for monitoring [26]. In addition, the effect of DLR on knee MRI regarding quantitative evaluation has not yet been studied, and the role of T2 values derived from T2 mapping with an acceleration factor of 2 and 3 using DLR has not been investigated.

The research gap we identified is the absence of prospective, comprehensive examination of DLR in knee MRI, particularly for its application accelerated, high-resolution T2 mapping. Crucially, it remains unknown if adding a T2 sequence to a DLR-accelerated protocol improves diagnostic performance for cartilage lesions, as prior studies lack evidence against an arthroscopic reference standard. Our study was designed to directly fill this gap. This study aimed to (1) compare image quality (both subjective and objective) of high in-plane spatial resolution FSE-MRI using CR or DLR, (2) examine the consistency of quantitative T2 values derived from T2 mapping with an acceleration factor of 2 or 3 using CR or DLR methods, and (3) evaluate whether the addition of a T2 mapping sequence to a routine MR imaging protocol using DLR could improve diagnostic performance in the detection of surgically confirmed cartilage lesions.

## Materials and methods

### Subjects

**Fig. 1:**
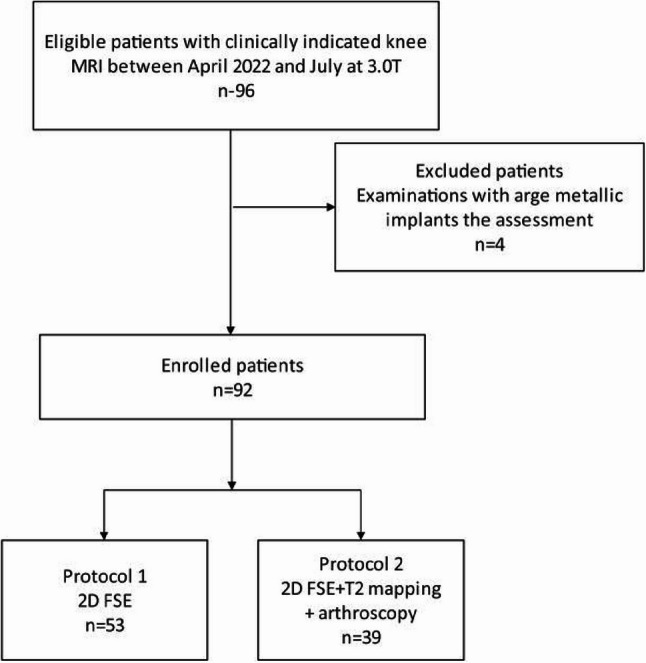
The workflow of subject inclusion and exclusion in this study

This prospective study was approved by the institutional review board of our hospital (Approval No.WDRY2022-K174) in accordance with the Declaration of Helsinki. A total of 96 adult patients (40 males and 56 females) signed informed consent before undergoing knee magnetic resonance examination from April 2022 to July 2022. Participants were consecutively recruited from the pool of institutional orthopedic and sports medicine outpatients with clinical MRI indications of acute or chronic knee pain, dysfunction, and instability were considered for eligibility. Exclusion criteria were general contraindications for MRI or incomplete study data. A final sample of 92 participants was included for analysis; 4 patients with arge metallic implants were removed; 39 of 92 patients who completed both 2D FSE sequences also a T2 mapping sequence (Fig. 1).

### MRI acquisition

A total of 96 subjects underwent an MR scan examination at the foot-first and supine position on a 3.0 Tesla MR scanner (Signa Architect, GE Healthcare, Milwaukee, WI) using 18-channel transmitter/receiver knee coil. The lower margin of all knee patella was positioned at the center of the MR scanner. The proposed rapid knee FSE MRI protocol was shown in Table 1. In order to examine the feasibility of T2 mapping with the acceleration factor of 3, 2D multi-echo FSE with the k-space based acceleration factor of 2 and 3 (i.e., auto calibrating reconstruction for cartesian sampling, ARC) was used to acquire T2 mapping (T2_ARC = 2_, T2_ARC = 3_). All acquired images were automatically generated as FSE_O_ (T1WI_O_, FS-T2WI_O_, FS-PDWI_O_, T2_O − ARC=2_ and T2_O − ARC=3_) and FSE_DLR_ (T1WI_DLR_, FS-T2WI_DLR_, FS-PDWI_DLR_, T2_DLR − ARC=2_ and T2_DLR − ARC=3_) respectively using both an inline CR algorithm and a DLR algorithm with high-strength noise removal feature (a commercial product name AIR™ Recon DL, GE Healthcare; hereinafter referred to DLR).The deeply optimized CNNs algorithm was embedded in the MR image reconstruction pipeline to directly output DLR-based images with less noise and truncation artifacts to achieve shorter scan time and higher image quality [27, 28]. The conventional and novel reconstruction for the accelerated 2D FSE sequences was performed using a standard parallel imaging (PI) method. Specifically, we utilized the k-space-based parallel imaging algorithm, autocalibrating reconstruction for Cartesian sampling (ARC). Due to unmet needs such as long acquisition time, only two patients were randomly selected to examine the difference of signal-to-noise ratio and time between the same imaging protocol using CR and DLR for verification.

**Table 1:** Sequences and parameters

	view	Field-of-view(mm^2^)	Voxel(mm^3^)	No.slice	Thickness(mm)	Gap(mm)	TR(ms)	TE(ms)	Acceleration factor	Nex	Scan time
PDWI-FS	sagittal	160 × 160	0.5 × 0.6 × 3.5	20	3.5	0.5	1799	35	2	1	1min27s
T1WI	sagittal	160 × 160	0.4 × 0.6 × 3.5	20	3.5	0.5	678	7.9	2	1	1min19s
PDWI-FS	coronal	160 × 160	0.4 × 0.6 × 3.5	20	3.5	0.5	1799	35	2	1	55s
T2WI-FS	axial	160 × 160	0.4 × 0.6 × 3.5	18	3.5	0.5	1799	35	2	1	1min8s
T2 mapping	sagittal	160 × 160	0.5 × 0.6 × 3.5	20	3	0	1500	8.464 ~ 67.712, Echo space = 3.96	2	1	7min6s
T2 mapping	sagittal	160 × 160	0.5 × 0.6 × 3.5	20	3	0	1500	8.464 ~ 67.712, Echo space = 3.96	3	1	5 min

*FS* Fat suppression, *PDWI* Proton density weighted image, *T1WI* T1-weighted image, *T2WI* T2-weighted image, *TR* Repetition time, *TE* Echo time

### MRI image analysis

Two musculoskeletal radiologists, with 5 (junior) and 15 (senior) years of experience respectively, independently assessed the routine MR images. Both readers were blinded to all patient information and reconstruction methods. To limit the potential for recall bias, interpretations of the conventional images (with and without DLR) were carried out at an interval of at least 2 weeks. Two radiologists independently assessed pathologies and internal derangement in the menisci, ligaments, and cartilage comprising six regions (patellar cartilage, PC; femoral trochlear cartilage, FTC; medial femoral condyle cartilage, MFC; lateral femoral condyle cartilage, LFC; medial tibial condyle cartilage, MTC; lateral tibial plateau cartilage, LTC) [29]. Structural abnormalities were graded as 0 = normal, 1 = altered (degenerative, postoperative), and 2 = tear. Cartilage defects were classified using a modified version of the classification system of the International Cartilage Repair Society (ICRS), as follows: Grade 0: Smooth cartilage surface, no defects, homogeneous signals, no abnormal signal in the subchondral bone; Grade I : Intact cartilage, smooth surface, no defect, focal abnormal signal; Grade II : Visible cartilage surface defect, defect depth < 50% of the full cartilage thickness; Grade III : The defect depth > 50% of the full cartilage thickness but not the whole cartilage. Grade IV : Complete loss of the whole layer cartilage with exposed subchondral bone [30]. When there were different scores of the injuried sites in the same part of cartilage, the highest score was recorded [2, 12, 31, 32]. Areas of bone marrow edema (femoral, patellar, tibial), as well as fractures and joint effusion, were evaluated being present or absent. All evaluated items of anatomic structures and pathologies are displayed in Supplementary Table S1.

T2 maps for the articular cartilage of the knee joint with a color scale ranging between 25 and 75 msec were generated immediately followed by the MR examination by a technologist using a postprocessing software on the advanced workstation (AW version 4.7, GE Healthcare).The senior radiologist measured the knee cartilage T2-relaxation time. ROIs excluding bone and fluid in the joint cavity were placed at above 6 regions of the articular cartilage. Each ROI was carefully sketched to ensure that at least 2 voxels of thickness were included in the vertical direction of the cartilage to exclude voxels in adjacent tissues. If there was a slight movement of the knee between scans, the images were aligned using rigid transformations to ensure that each sequence represented the same physical location. T2 measurements of each ROI were averaged for each sequence.The mean and SD of T2 relaxation time for each ROI were recorded. Only cartilaged with Grade 0-III were included to measure T2 values due to loss of the whole Grade-IV osteoarthritis cartilage and barity of subchondral bone. In the case of multiple lesions along the same articular surface, the largest lesion was drawed.

### Subjective and objective assessment of FSE image quality

The subjective assessment of knee on images were evaluated for overall image quality, artifacts, sharpness using a 5-point Likert scale (1 = non-diagnostic; 2 = low image quality; 3 = moderate image quality; 4 = good image quality; 5 = excellent image quality) of PDWI_O_, PDWI_DLR_, T1WI_O_ and T1WI_DLR_ images [2].

Circular regions of interest (ROIs) with an area of 16 pixels were respectively positioned at the bone marrow cavity, femur-side cartilage, synovial fluid, infrapatellar fat pad, and anterior cruciate ligament (ACL) of each knee and repeated for three times to obtain an average signal intensity (SI) for each ROI. The standard deviation (SD) of background noise with the same-size ROI was also measured for three times and averaged. Signal-to-noise ratio (SNR) and contrastion-to-noise ratio (CNR) of each target was calculated separately with the following formulas:

$$\mathrm{SNR} = \mathrm{SI}_\mathrm{cartilage} /\mathrm{SD}_\mathrm{background}$$

$$\mathrm{CNR} = \mid\mathrm{SI}_\mathrm{cartilage}-\mathrm{SI}_\text{superficial fat}\mid/\mathrm{SD}_\mathrm{background}$$

where SI and SD accordingly represents mean signal intensity and standard deviation of signal intensity.

### Arthroscopic diagnosis

Surgery was performed by orthopedic surgeons (with 15 years of experience in knee arthroscopy) who had the original MRI interpretations available at the time of surgery.All articular surfaces were evaluated intraoperatively by the same orthopedic surgeon according to International Cartilage Repair Society (ICRS) classification. ICRS scores ranged from Grade 0 to Grade 4 (Grade 0, normal articular cartilage; Grade I, inhomogeous, surface intact, cartilage swelling; Grade II, superficial ulceration, fissuring, fibrillation, cartilage thinning > 50%; Grade III, ulceration, fissuring, fibrillation; >50% of depth of cartilage; Grade IV, fully deprived cartilage with exposed subchondral bone) [30]. In the case of multiple lesions along the same articular surface, the largest lesion was scored.

### MRI diagnostic efficacy of knee cartilage injury

The diagnostic performance of FSE-based images using CR and DLR on differentiation of knee cartilage with different ICRS scores (Grade 0 and Grade I-III was analyzed using arthroscopy as standard reference.

To examine the diagnostic performance, the mean T2 values for six subregions (PC, FTC, MFC, LFC, MTC and LTC) were calculated and compared between each two groups. The T2 maps were then used to detect areas of increased T2 relaxation time on articular surfaces that appeared normal with the routine MR imaging protocol. Articular cartilage that appeared normal with the routine MR imaging protocol but showed increased T2 relaxation time on the T2 maps was classified as a grade 1 cartilage lesion [10]. When a grade 1 cartilage lesion was identified on the T2 maps, the presence or absence of four features of the area of increased T2 relaxation time was determined. These features included whether the area of increased T2 relaxation time (a) was two or more color scales higher than normal, (b) was more than 1 cm in maximal diameter, (c) was present on at least two consecutive images, and (d) involved the entire thickness of the deep cartilage layer [33, 34].

### Statistics analysis

Data analysis was conducted according to previous studies that separately evaluated the diagnostic capabilities of a 2D FSE sequence for comprehensive knee MRI and the clinical value of adding a T2 mapping sequence to the routine knee MRI protocol, respectively [2, 10, 35].

Analysis was conducted using SPSS software (IBM, version 25.0). Data presented in mean ± standard deviation ($\overline{\mathrm x}$ ± s) or median ± interquartile ranges (M ± IQ) according to normal distribution that was analyzed by Shpiro-Wilk test. Categorial variables (subjective evaluation: overall image quality, sharpness, diagnosis performance) or continuous variables (objective evaluation: SNR, CNR) were analyzed. Inter-reader agreement of each pathology and image quality scores was assessed by using Cohen’s κ with 95% confidence intervals and interpreted as follows: 0.20 or less, poor agreement; 0.21–0.40, fair agreement; 0.41–0.60, moderate agreement; 0.61–0.80, substantial agreement; and greater than 0.80, almost perfect agreement. The latter was analyzed using paired t test or Wilcoxon rank sum test according to data distribution normality and variance equality. The statistical analysis for this section was conducted on the full cohort of 92 subjects.

Paired t Test or Wilcoxon signed-rank test was used to examine difference of ICRS scores and mean and SD values of T2 measurements after normal distribution and variance equality was respectively confirmed by Shapiro-Wilk test and Levene test. The Bland-Altman analysis were used to evaluate the correlation and agreement between T2 values derived from each image dataset. Receiver operating characteristic (ROC) curve was used to analyze the diagnostic performance of FSE_O_, FSE_DLR_ and T2_O − ARC=2,3_/T2_DLR − ARC=2,3_ in differentiation of groups using 39 cases with arthroscopic results as reference. Delong test was used to compare the area under ROC curves (AUCs). *P* < 0.05 was considered statistically significant. All comparisons were performed on a sub-cohort of 39 subjects.

## Results

### Patient demographics

A total of 92 participants (38 males, 54 females) at the age of 18–82 years (mean age 48.4 ± 15.4 years old) with BMI of 18.2–29.98 kg/m^2^ (average BMI of 23.6 kg/m^2^) were finally included in our study. There were knee osteoarthritis (KOA) (62 cases, 67.4%), trauma (16 cases, 17.4%), tumor (3 cases, 3.3%), anterior cruciate ligament reconstruction (3 cases, 3.3%), and synovitis (8 cases, 8.6%). (Supplementary Figure S1a). As the T2 mapping followed by the routine protocol prolonged the total scan time, some patients who cannot tolerate long scan time dropped out during examination. Finally 39 patients with knee degenerative changes also underwent a T2 mapping scan and received an arthroscopy exam at an average interval of 22 ± 5.3 days. Totally, 234 measurements for 6 regions of each cartilage in a total of 39 cases were identified and graded, including 105 Grade 0 (44.8%), 35 Grade I (14.9%), 40 Grade II (17.1%), 44 Grade III (18.9%) and 10 Grade IV (4.3%). (Supplementary Figure S1b).

### Subjective and objective image analysis

**Fig. 2:**
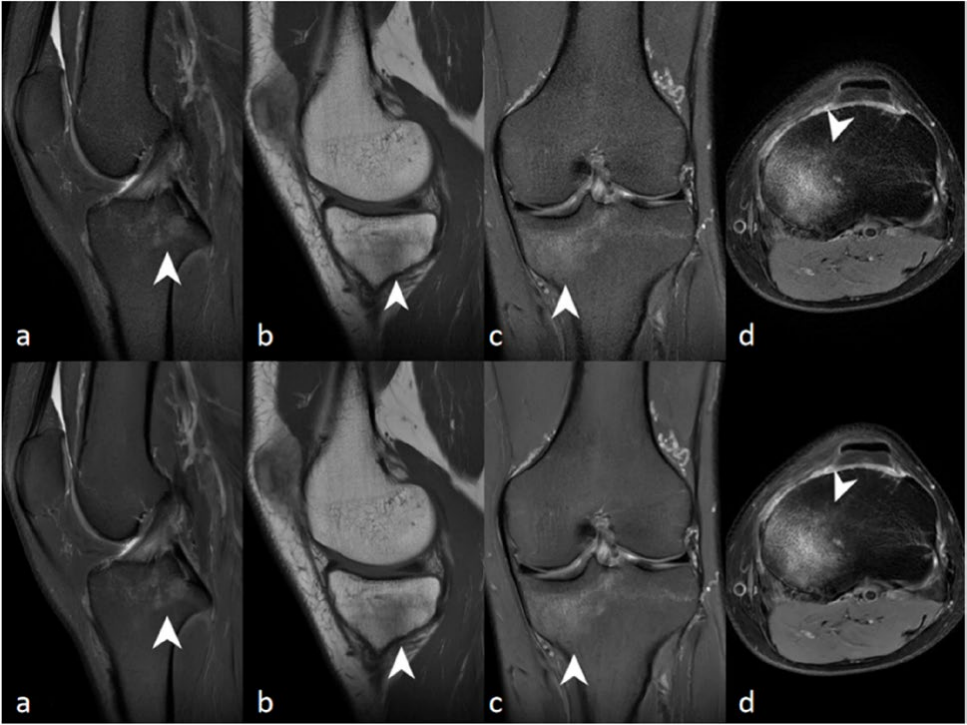
A 31-year-old patient with pain in the medial side of the left knee after trauma. The original convetional reconstruction (upper row) and deep learning-based reconstruction (lower row) (**a**, **c**) fat-suppression PDWI, **b **T1WI, **d** fat-suppression T2WI. FSE_DLR_ showed higher image quality with less noise and better clarity of the anatomic structures. Note that bone marrow edema (white arrowheads) of the tibial plateau is clearly definable on both FSE_O_ and FSE_DLR_

Inter-reader agreement of subjective assessment ranged from 0.70 (95% CI: 0.46–0.94,) to 0.89 (95% CI: 0.79–0.99,), (Table 2), and higher score on FSE_DLR_ than FSE_O_. Sharpness for FSE_DLR_ was rated to be excellent (median Likert score, 5; range, 5–5), higher compared to FSE_O_ (median Likert score, 5; range, 4–5),(*P* < 0.001)). Both SNR and CNR of FSE_DLR_ were higher than those of FSE_O_ (*P* < 0.001). FSE_DLR_ showed significantly higher SNR of LFC, LFC cartilage, synovial fluid and subpatella fat pad, and CNR of cartilage/synovial fluid than FSE_O_ (*P* < 0.001). (Fig. 2; Table 3, Supplementary Figure S2).

**Table 2:** Inter-reader consistency of image quality on FSE_O_ and FSE_DLR_

items	Reader 1			Reader 2			Cohen’s κ	
	FSE_O_	FSE_DLR_	*P* value	FSE_O_	FSE_DLR_	*P* value	FSE_O_	FSE_DLR_
Over all image qulaity	4.90[5(5,5)]	4.95[5(5,5)]	0.206	4.85[5(5,5)]	4.91[5(5,5)]	0.083	0.75(0.54–0.95)	0.75(0.48–0.99)
Sharpness	4.68[5(4,5)]	4.89[5(5,5)]	<0.001	4.71[5(4,5)]	4.88[5(5,5)]	<0.001	0.89(0.79–0.99)	0.83(0.66–0.99)
Diagnosis confidence	4.91[5(5,5)]	4.96[5(5,5)]	0.739	4.88[5(5,5)]	4.95[5(5,5)]	0.617	0.70(0.46–0.94)	0.88(0.65–0.99)

The results are reported as mean [median (interquartile range)]

**Table 3:** Comparison of SNR and CNR in different tissues between PDWI_O_, T1WI_O_ images and PDWI_DLR_, T1WI_DLR_ images

	position	PDWI_O_	PDWI_DLR_	*P* value	t/z value	T1WI_O_	T1WI_DLR_	*P* value	t/z value
SNR	lateral femoral condyle	56.4(49.3)	215.1(177.9)	<0.001	−5.841**	398.4(257.7)	972.6(814.8)	<0.001	−5.841**
	cartilage of lateral femoral condyle	144.8 ± 53.6	530.8 ± 199.3	<0.001	17.059*	110.7(81.3)	339.3(274.5)	<0.001	−5.841**
	synovial fluid	353.2 ± 146.9	1287.5 ± 533.5	<0.001	15.635*	113.3(85.7)	344.2(279.2)	<0.001	−5.841**
	subpatellar fat pad	83.6(53.7)	268.6(200.4)	<0.001	−5.841**	374.7(240.7)	1068.7(834.9)	<0.001	−5.841**
	anterior cruciate ligament	75.5(74.1)	273.8(333.0)	<0.001	−5.841**	100.4(65.8)	308.2(212.2)	<0.001	−5.841**
CNR	cartilage/synovial fluid	208.4 ± 99.2	756.7 ± 356.1	<0.001	13.878*	7.1(9.4)	23.4(36.6)	<0.001	−5.830**

*PDWI* Proton density weighted imaging, *T1WI* T1 weighted imaging, *DLR* Deep learning reconstruction, *SNR* Signal-to-noise ratio, *CNR* Contrast-to-noise ratio. * t value, ** z value

Concerning the detection of degeneration or tears of the menisci and ligaments, inter- and intra-reader agreement was almost perfect, withκvalues between 0.94 (95% CI: 0.85–1.0.85.0) and 1.00 (95% CI: 1.0–1.0). There was no clinically relevant difference concerning the detection of structural abnormalities between FSE_O_ and FSE_DLR_. Regarding the detection and evaluation of cartilage defects, inter- and intra-reader agreement was substantial to almost perfect with κ values between 0.70 (95% CI:0.52–0.88) and 0.93 (95% CI:0.85–1.0.85.0). No difference was found between the readers and the two sequences FSE_O_ and FSE_DLR_ with regard to the detection of bone marrow edema and joint effusion. Intra- and inter-reader agreement of detected pathologies is summarized in Supplementary Table S2.

### T2 measurements

**Fig. 3:**
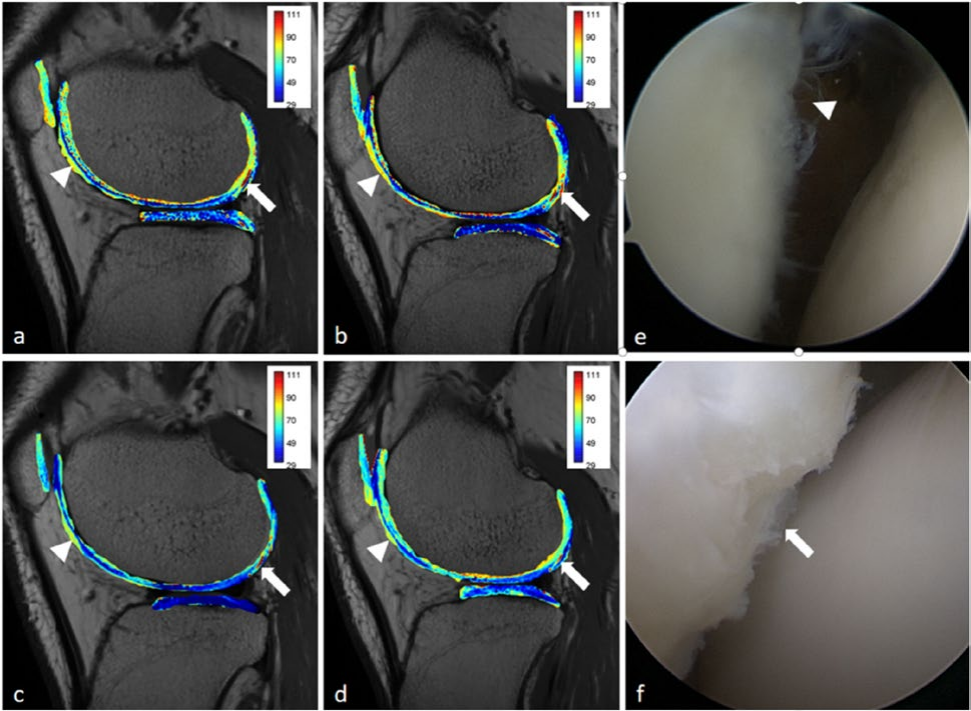
T2 mapping with the acceleration factor of 2 and 3 using CR and DLR (**a**, T2_O − ARC=2_; **b**, T2_O − ARC=3_; **c**, T2_DLR − ARC=2_; **d**, T2_DLR − ARC=3_) showed the lesion site of cartilages with ICRS Grade I (white arrowhead) and the lesion sites of cartilages with ICRS Grade II (white arrow) respectively corresponding to (**e**, **f**) arthroscopic results

All T2 values conformed to normal distribution and there were significant differences between T2 _O−ARC=3_ and T2_DLR − ARC=3_ in patellar cartilage, betweenT2_O − ARC=2_ and T2_DLR − ARC=3_ in femoral cartilage, between T2_DLR − ARC=2_ and T2_DLR − ARC=3_ as well as between T2_O − ARC=2_ and T2_DLR − ARC=3_ in tibial cartilage (all *P* < 0.05) (Supplementary Figure S2). T2 relaxation time significantly increased with the severity of morphological cartilage injury (Fig. 3, Supplementary Table S3).

### Consistency and diagnostic efficacy of routine FSE images and T2 relaxation time

**Fig. 4:**
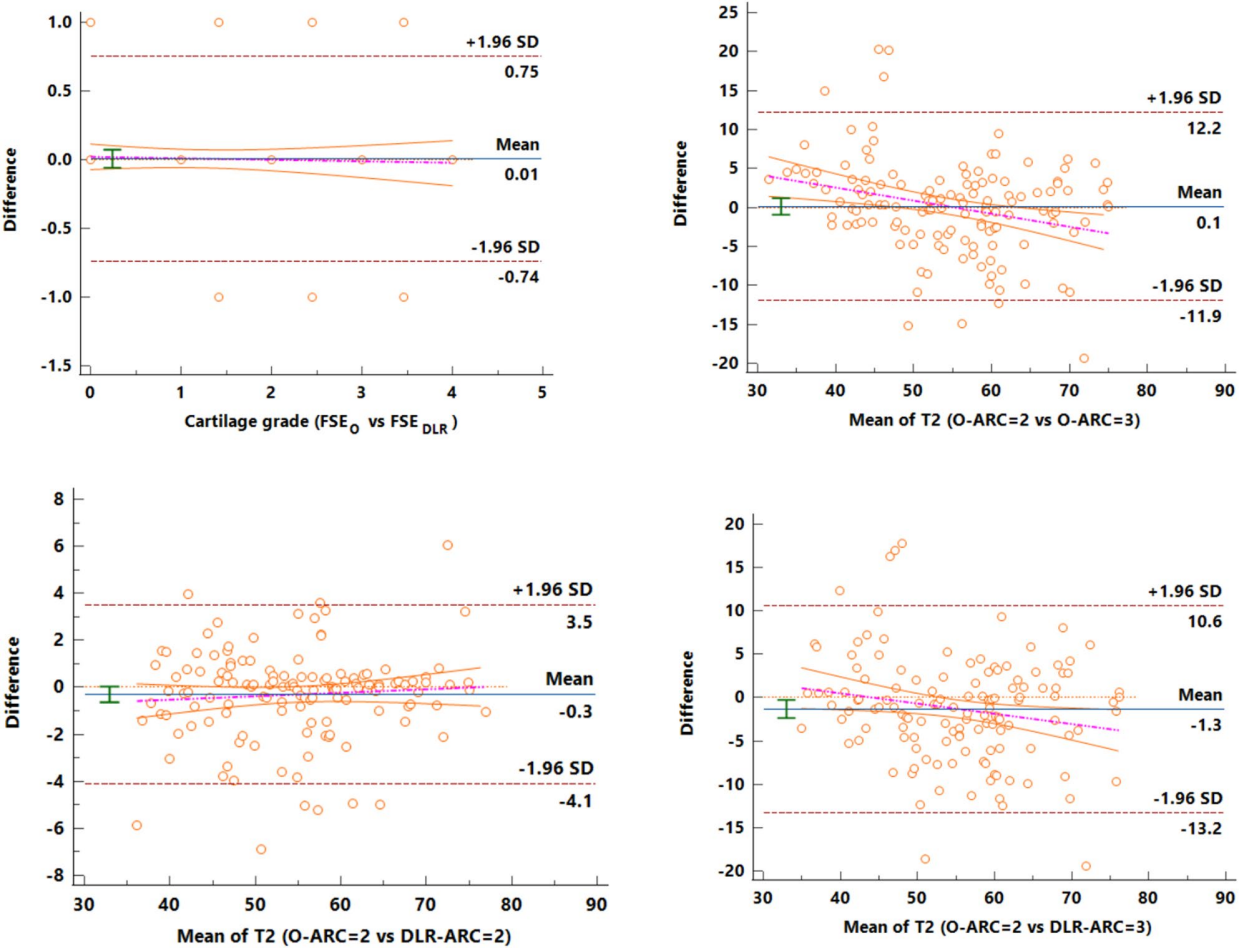
Bland-Altman plots present the consistency of knee cartilage injury level classified based on routine FSE_O_ (κ = 0.83, 95% CI: 0.73–0.89) and FSE_DLR_ (κ = 0.88, 95%CI: 0.79–0.93) as well as based on T2_O − ARC_=3 (ICC = 0.85, 95% CI: 0.22–2.08), T2_DLR − ARC_=2 (ICC = 0.98, 95% CI: 0.10–0.62), T2_DLR − ARC_=3 (ICC = 0.82, 95% CI: 1.59–3.45) using T2_O − ARC_=2 as reference

In Bland-Altman plots, the agreement of knee cartilage injury level that was classified by two radiologists was moderate to high based on routine FSE_O_ (κ = 0.83, 95% CI: 0.73–0.89) and FSE_DLR_ (κ = 0.88, 95%CI: 0.79–0.93). The consistency of T2 value was moderate to high on T2_O − ARC=3_ (ICC = 0.85, 95% CI: 0.22–2.08), T2_DLR − ARC=2_ (ICC = 0.98, 95% CI: 0.10–0.62), T2_DLR − ARC=3_ (ICC = 0.82, 95% CI: 1.59–3.45) using T2_O − ARC=2_ as reference (Fig. 4).

**Fig. 5:**
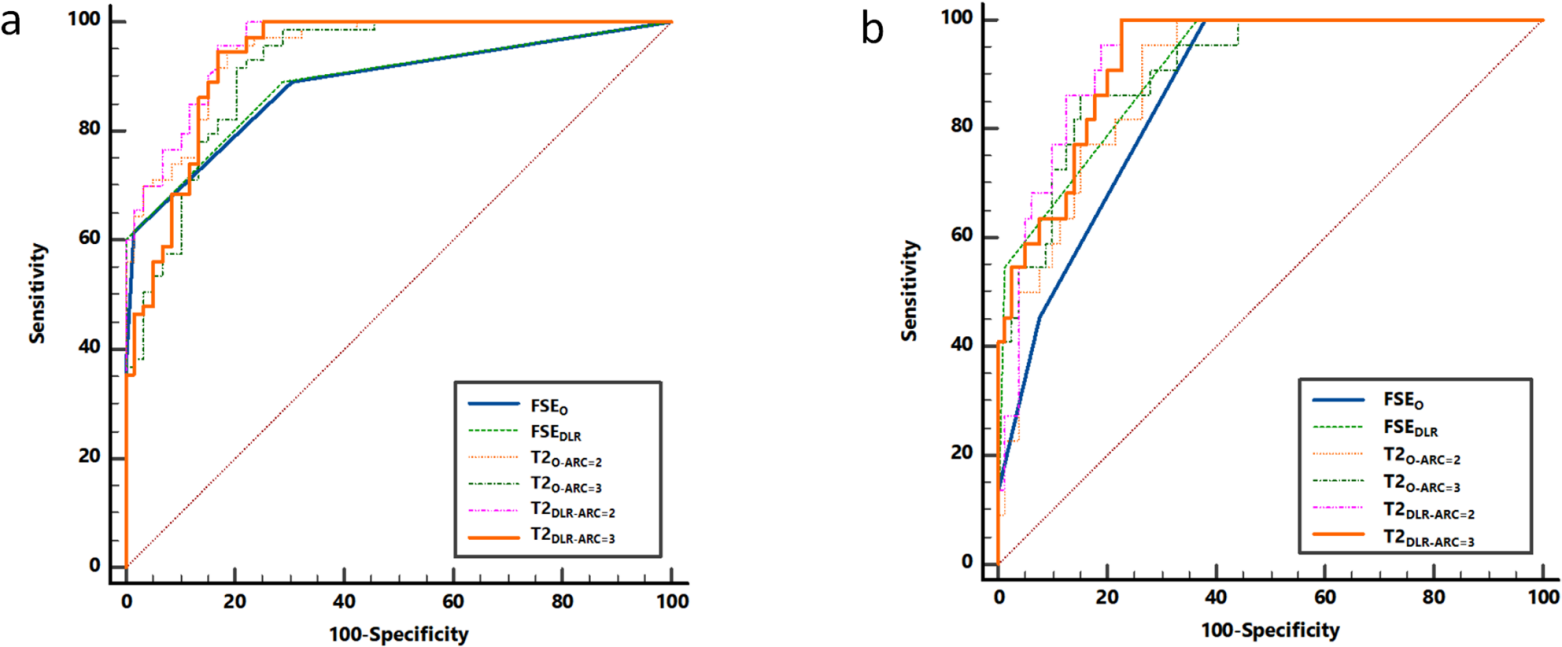
Area under the curves of cartilage diagnosis using different image information with and without DLR in differentiating (**a**) classification I group A (ICRS Grade 0) from group B (ICRS Grade I-III) as and (**b**) classification B: group A (ICRS Grade 0-I) from group B (ICRS Grade II)

In terms of diagnostic efficacy of T2 measurement, a total of 234 measurements were divided into two groups using two different classifications: classification I for group A (ICRS Grade 0) and group B (ICRS Grade I-III) and classification II for group A (ICRS Grade 0-I) and group B (ICRS Grade II). ROC curves showed that T2_DLR − ARC=2_ as well as T2_DLR − ARC=2_ and T2_DLR − ARC=3_ respectively had the highest diagnostic efficacy for classification A and B (Fig. 5, Supplementary Table S4-5); Delong test demonstrated there were statistically significant differences of T2_O − ARC=2_ vs. FSE_O_, T2_O − ARC=2_ vs. FSE_DLR_, T2_O − ARC=3_ vs. T2_DLR − ARC=2_ vs. FSE_O_, T2_DLR − ARC=2_ vs. FSE_DLR_ (*P* < 0.05, Supplementary Table S4, Supplementary Table S5). FSE_DLR_ (Reader 1 AUC, 0.77; 95% CI: 0.69–0.84 and Reader 2 AUC, 0.86; 95% CI: 0.78–0.91) had higher diagnostic performance than FSE_O_ (Reader 1 AUC, 0.74; 95% CI: 0.66–0.81 and Reader 2 AUC, 0.80; 95% CI: 0.72–0.86; *P* = 0.005) for articular cartilage lesions. Moreover, Reader 1 achieved the higher diagnostic efficacy (AUC, 0.84; 95% CI: 0.76–0.90) in differentiating normal-appearing from injury-visible cartilage when using both routine FSE_DLR_ images and T2_DLR − ARC=2,_while Reader 2 achieved an AUC of 0.86 (95% CI: 0.78–0.91) with routine FSE_DLR_ images. (Table 4).

**Table 4:** Comparison of the diagnostic performance for cartilage lesions between FSE_O_, FSE_DLR_ and T2_O − ARC=2,3_/T2_DLR − ARC=2,3_

sequence	FSE_O_	FSE_DLR_	FSE_O_+T2_O − ARC=2_	FSE_DLR_+T2_DLR − ARC=2_	FSE_O_+T2_O − ARC=3_	FSE_DLR_+T2_DLR − ARC=3_
Reader 1	0.74(0.66–0.81)^a^	0.77(0.69–0.84)^a^	0.75(0.67–0.83)^b^	0.84(0.76–0.90)^b^	0.77(0.69–0.84)	0.81(0.73–0.87)
Reader 2	0.80(0.72–0.86)	0.86(0.78–0.91)^c^	0.83(0.75–0.89)^c^	0.85(0.77–0.90)	0.82(0.75–0.88)	0.81(0.74–0.88)

Data are reported as AUCs (95% CI)

^a, b^ Significant difference between FSE_O_ and FSE_DLR_, as well as between FSE_O_+T2_O − ARC=2_ and FSE_DLR_+T2_DLR − ARC=2_ for Reader 1,*P* < 0.05

^C^ Significant difference between FSE_DLR_ and FSE_O_+T2_O − ARC=2_ for Reader 2,*P* < 0.05

## Discussion

We firstly proposed a feasible rapid high-resolution knee 2D FSE-based imaging protocol including T2 mapping using an inline deep learning-based reconstruction, and its diagnostic efficacy was elevated closer to arthroscopic results. Moreover, two radiologists had equivalent diagnostic efficacy on differentiating cartilages with Grade 0 from those with Grade I-IV when using both anatomical images and quantitative information. Notedly, DLR-based FSE images showed superior image quality to conventional reconstruction images and simultaneously increased sensitivity of lesion detection.

Diagnostic efficacy for knee ICRS grading was preserved or even improved using the DLR parallel-acquisition image sets. For instance, the sagittal PDWI sequence illustrated substantial scan time reduction. PDWI_DLR_ with acquisition time of 1’27’’ yielded approximately 4-time the SNR of PDWI_O_. Achieving a comparable SNR with PDWI_O_ required 8 excitations, extending the scan time went to 8’57’’. While parallel imaging reduces phase-encoding steps and scan time that potentially minimizes motion artifacts, it traditionally compromises SNR and introduces noise enhancement and residual aliasing due to reconstruction limitations [2, 35, 36]. Consistent with proper DLR studies in peripheral nerve and musculoskeletal imaging (e.g. knee) [2, 22, 27, 37–40] our DLR-based images demonstrated superior subjective and objective quality (e.g., higher sharpness, SNR in key structures like the lateral femoral condyle, lateral femoral condyle cartilage, synovial fluid and subpatellar fat pad, and improved CNR in cartilage/synovial fluid) compared to conventional reconstructions. Moreover, FSE_DLR_ showed higher diagnosis accuracy for Grade 0–3 cartilage injuries than FSE_O_, suggesting it may be a promising approach for clinical diagnosis of cartilage abnormalities.

T2 knee mapping had higher sensitivity to mild defects (I.e., cartilages with Grad 1–2) on knee than routine structural MR images. Conventionally, T2 mapping using parallel imaging with the acceleration factor of 2 has been widely utilized in research of degenerated musculoskeletal system (e.g., cervical spine, lumbar spine, knee, shoulder). The acquisition time of T2 mapping with the resolution of 0.3125 × 0.3125 × 3.5 mm^3^ respectively using an acceleration factor of 2 and 3 was 7’6’’ and 5’. Conventional reconstruction is restricted by data sampling-related factors (i.e., scan time, SNR, resolution) while DLR breaks the solid concept of triangle balance and offers both structural and quantitative images with less noise and Gib’’s artifact. In consistent with previous research, the mean T2 relaxatory value at the range of 46.6 ± 6.1ms (Grade 0) to 65.9 ± 7.3ms (Grade III) varied significantly with the severity of morphological cartilage defect. Knee T2 relaxation time using an acceleration factor of 2 using both CR and DLR algorithms showed high reproducibility and no statistically different to that using an acceleration factor of 3, but the latter had lower SNR. For both T2 measurements of subjects, T2_DLR − ARC=2_ and T2_DLR − ARC=3_ respectively had significantly smaller SD than T2_O − ARC=2_ and T_O−ARC=3_ along with the smallest SD of T2_DLR − ARC=2_ among all T2 measurements. Additionally, there were significant differences between T2_DLR − ARC=3_ and T2_O − ARC=3_ of patella cartilage, T2_DLR − ARC=3_ and T2_O − ARC=2_ of femur cartilage, T2_DLR − ARC=2_ and T2_DLR − ARC=3_ as well as T2_DLR − ARC=3_ and T2_O − ARC=2_ of tibial cartilage. That is, more missing detailed information was observed when a higher acceleration factor was used; T2_DLR − ARC=3_ had the poorest reliability. Thus, the statistical difference between T2_DLR − ARC=3_ and T2_O − ARC=3_ of patella cartilage might attribute to field susceptibility, magic angle effect, partial volume effect caused by the interface between cartilage and synovial fluid, and other factors [41–43]. Noteworthy, there was even no statistically significant difference of AUC among the four groups despite slightly higher diagnostic performance of T2_DLR − ARC=2_ on cartilage lesion classification than T2_O − ARC=3_. Overall, T2_DLR − ARC=3_ showed no different detection rate and diagnosis of cartilage lesions to any other T2 measurements, indicating T2 mapping with an acceleration factor of 3 could be an acceptable and compromised option for clinical demand especially for subjects with poor tolerance or compliance.

This study firstly reported diagnostic accuracy imporved when readers evaluated knee cartilage using a combined assessment of routine FSE images and T2 mapping with deep learning reconstruction algorithm, based on prospectively defined cutoff values. This undersocres the clinical potential of this optimized protocol. By providing rapid, high-quality anatomic and quantitative data with increased SNR, rapid, diagnostic efficacy can be enhanced. The total scan time for T2 mapping with the acceleration factor of 3 was reduced by 42%, without a significant loss in diagnostic performance (e.g., T2 mapping with the acceleration factor of 3 and 2 (T2_DLR − ARC=2_ and T2_DLR − ARC=3_ respectively with AUC of 0.93 (95% CI: 0.79–0.98) and 0.93 (95% CI: 0.76–0.96) for classification A as well as 0.95(95% CI: 0.81–0.98) and 0.92(95% CI: 0.79–0.96) for classification B). The junior radiologist (Reader 1) showed improved performance in differentiating Grade 0 from Grade I/II cartilage lesions using the combined protocol, consistent with previous studies where quantitative data aids less experienced readers [10]. In contrast, the senior radiologist (Reader 2), whose their diagnostic accuracy using structural images alone was already high, derived no additional benefit, suggesting T2 mapping could be selsectively employed. Therefore, adding T2 mapping to the routine 2D FSE protocol may improved diagnostic confidence for junior radiologists, while senior radiologists can optimize workflow by using the routine protocol alone. Reduced scan time lowers costs, improves patient comfort and compliance, potentially reduces motion artifacts, and may facilitate earlier diagnosis [12, 13].

Our study has several limitations. First, the study population is relatively small and only acquired on a 3.0-T MRI scanner from a single institution, confining our results to a specific magnetic field strength and limiting generalization. Prospective, multi-center validation with larger cohorts is essential. Second, the evaluation of knee cartilage injury was based on a comparison between conventional and DLR-reconstructed images, including T2 mapping. Our focus was limited to articular cartilage because T2 relaxation time is a recognized biomarker for knee osteoarthritis [44], other associated features such as meniscal pathology, bone marrow lesions, and peripheral muscles were not assessed and warrant future study. Third, despite higher inter- and intra-observer agreement for cartilage segmentation on T2 maps, quantitative analysis was performed by a single radiologist. Fourth, as a cross-sectional study, the lack of longitudinal data may limit conclusions regarding T2 value changes over time or after intervention. Assessing the sensitivity of the T2 relaxation time in long-term follow-ups is crucial. The last but not the least, the use of high-resolution T2 mapping (0.5 × 0.6 mm^2^) may have contributed to case loss and longer acquisition; employing a lower-resolution T2 mapping (e.g., 0.8 × 0.8 mm^2^) with DLR’s denoising advantages could be explored to balance diagnostic efficacy and practicality.

In conclusion, a rapid, high-resolution protocol combinging 2D FSE structural imaging and T2 mapping using deep learning reconstruction is feasible. It provides improved image quality with fewer artifacts and shos potential to enhance diagnostic efficacy for cartilage lesions, particularly for less experienced radiologists, with a clinically acceptable scan time under 10 min. These preliminary findings suggests its potential clinical value; however, further validation with larger, larger, multi-center cohorts is the necessary a next step to confirm its utility and generalizability.

## Supplementary Information

Supplementary Material 1.

## Data Availability

The datasets generated or analyzed during the study are available from the corresponding author on reasonable request.
