# Supplementary material for: Prospective study assessing the validity of accelerated 2D Fast Spin Echo (2D FSE) based high-resolution knee MRI and T2 mapping using deep learning reconstruction
Source: BMC Musculoskelet Disord. 2026 Jan 15;27:125. doi: 10.1186/s12891-025-09482-2 (PMC12892454; doi:10.1186/s12891-025-09482-2)
Supplement: Supplementary file 1 — Supplementary Material 1. [file 12891_2025_9482_MOESM1_ESM.zip › Supplemental Material/Supplementary Table and figure legends.docx]

Supplementary Fig. S1 Box plots for T2 measurements on T2 mapping with acceleration factor of 2 or 3 using conventional and deep learning-based reconstruction algorithm.

Supplementary Table S1 Evaluated items of anatomic structures and pathologies of the knee

Supplementary Table S2 Intra- and inter-reader agreement of detected pathologies in FSE_O_ and FSE_DLR_ imaging

Cohen’sκ(with a 95% confidence interval) Intra- and inter-reader agreement of each pathology between FSE_O_ and FSE_DLR_

Supplementary Table S3 Mean T2 relaxation values (ms) of cartilage with different ICRS level.

Supplementary Table S4 Sensitivity, specificity and diagnosis cutoff of FSE-based sequences for classification A.

Supplementary Table S5 Sensitivity, specificity and diagnosis cutoff of FSE-based sequences for classification B.
