# Supplementary figures and images for: Prospective study assessing the validity of accelerated 2D Fast Spin Echo (2D FSE) based high-resolution knee MRI and T2 mapping using deep learning reconstruction
Source: BMC Musculoskelet Disord. 2026 Jan 15;27:125. doi: 10.1186/s12891-025-09482-2 (PMC12892454; doi:10.1186/s12891-025-09482-2)

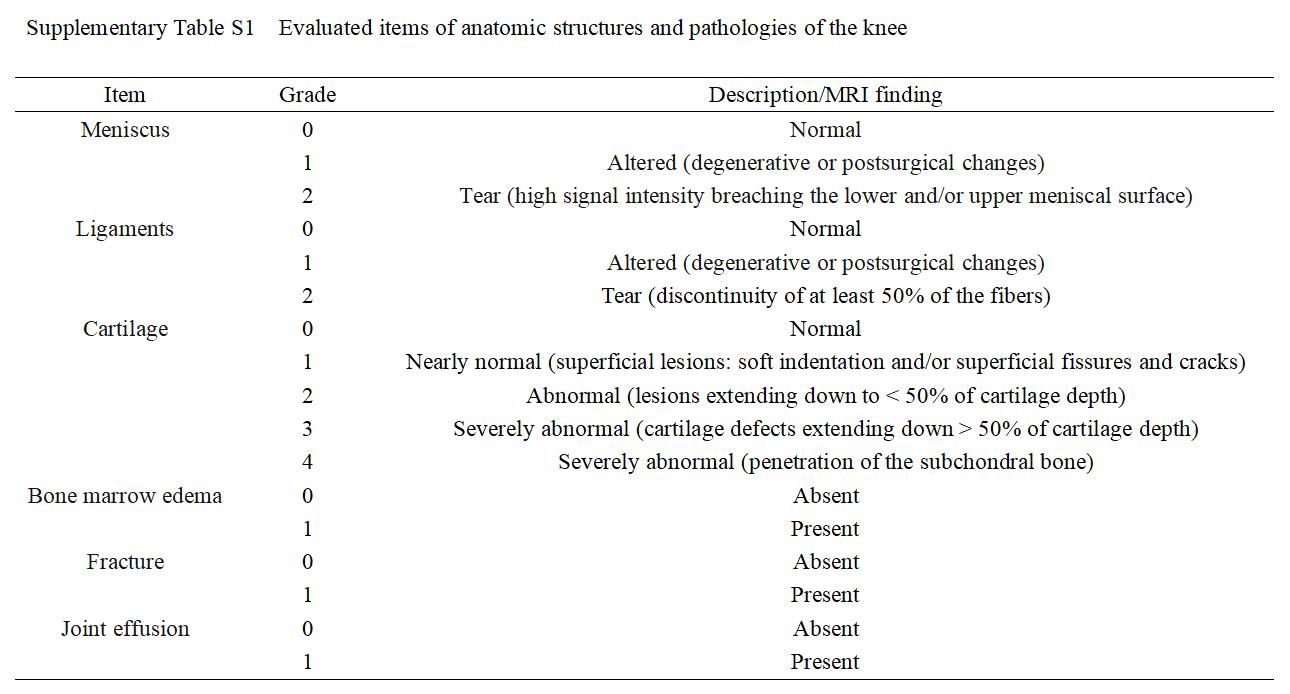

Supplement: Supplementary file 1 — Supplementary Material 1. [file 12891_2025_9482_MOESM1_ESM.zip › Supplemental Material/Supplemental Table S1.jpg]

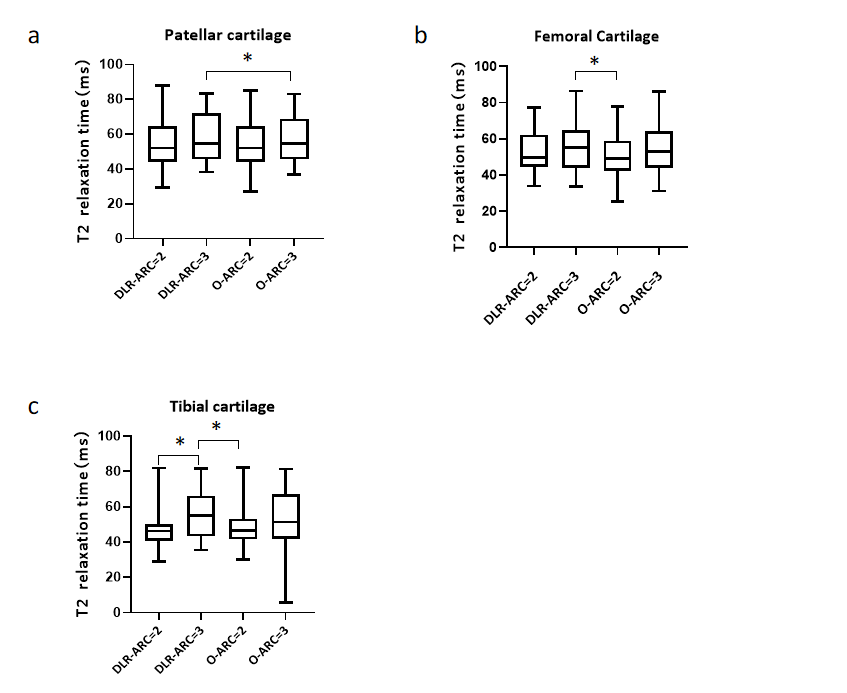

Supplement: Supplementary file 1 — Supplementary Material 1. [file 12891_2025_9482_MOESM1_ESM.zip › Supplemental Material/Supplementary Fig. S2.png]

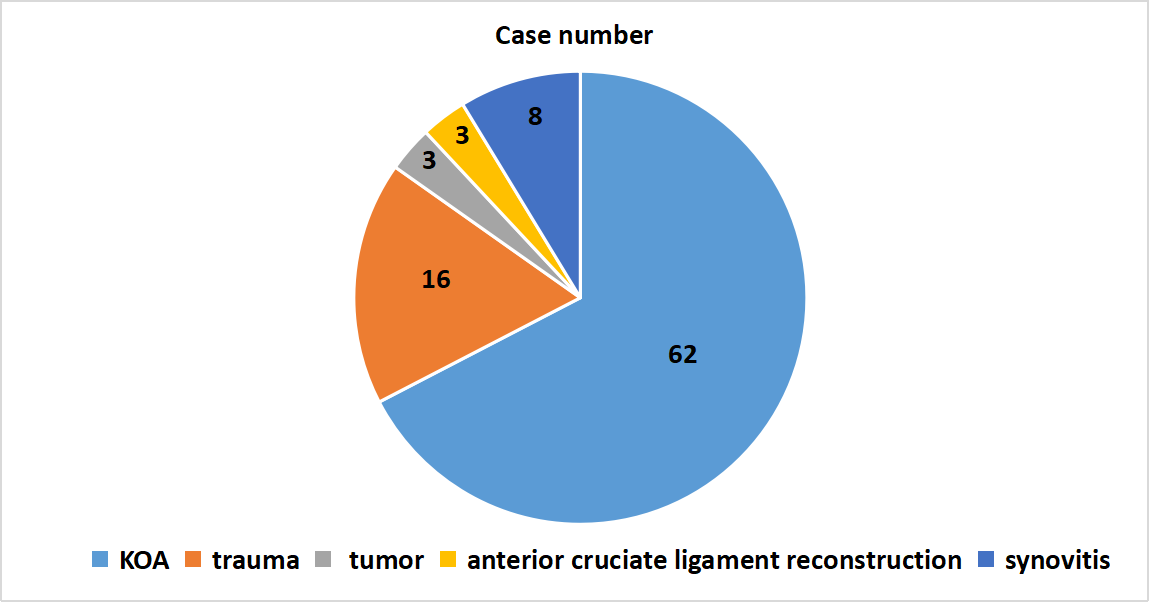

Supplement: Supplementary file 1 — Supplementary Material 1. [file 12891_2025_9482_MOESM1_ESM.zip › Supplemental Material/Supplementary Fig.S1a.png]

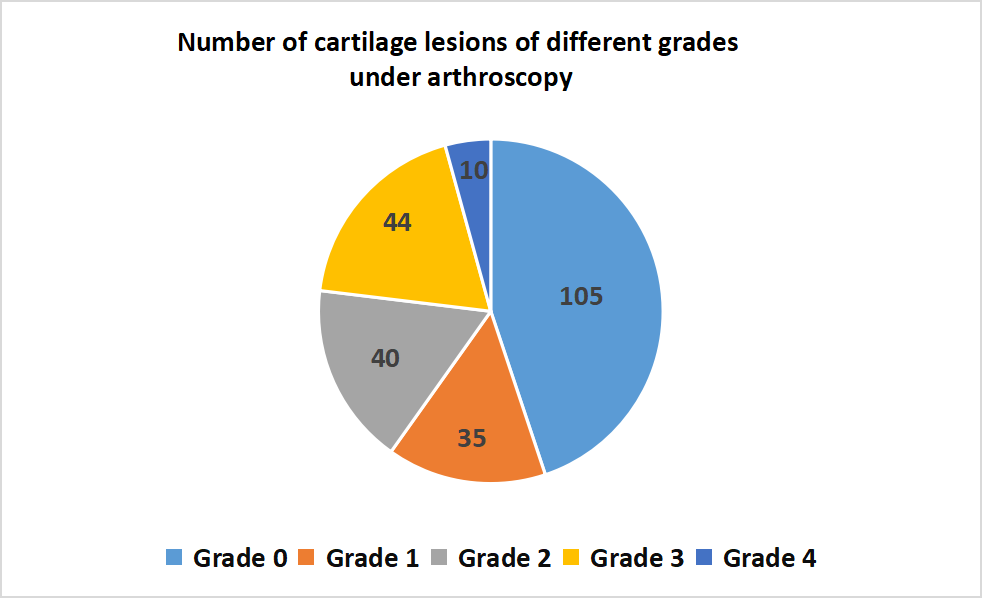

Supplement: Supplementary file 1 — Supplementary Material 1. [file 12891_2025_9482_MOESM1_ESM.zip › Supplemental Material/Supplementary Fig.S1b.png]

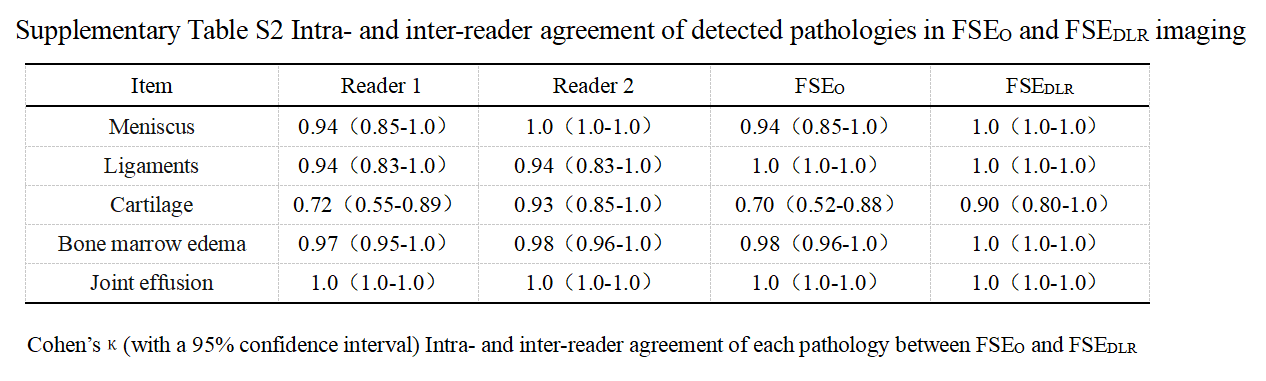

Supplement: Supplementary file 1 — Supplementary Material 1. [file 12891_2025_9482_MOESM1_ESM.zip › Supplemental Material/Supplementary Table S2.png]

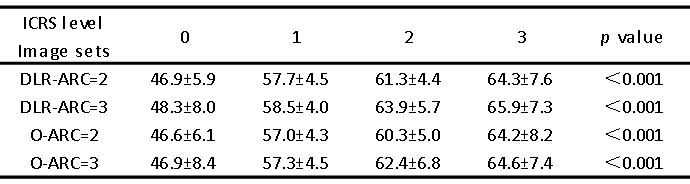

Supplement: Supplementary file 1 — Supplementary Material 1. [file 12891_2025_9482_MOESM1_ESM.zip › Supplemental Material/Supplementary Table S3.png]

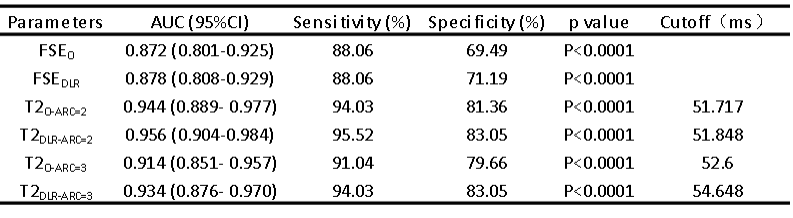

Supplement: Supplementary file 1 — Supplementary Material 1. [file 12891_2025_9482_MOESM1_ESM.zip › Supplemental Material/Supplementary Table S4.png]

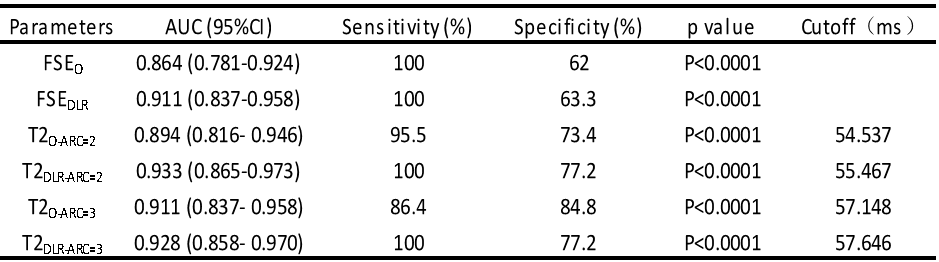

Supplement: Supplementary file 1 — Supplementary Material 1. [file 12891_2025_9482_MOESM1_ESM.zip › Supplemental Material/Supplementary Table S5.png]

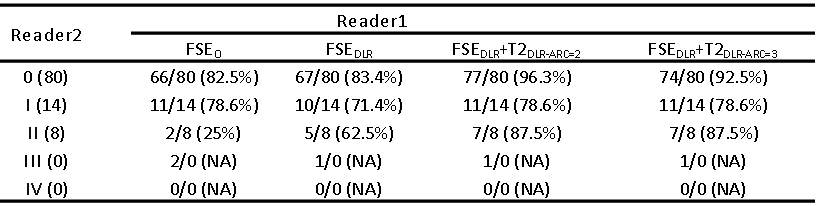

Supplement: Supplementary file 1 — Supplementary Material 1. [file 12891_2025_9482_MOESM1_ESM.zip › Supplemental Material/Supplementary Table S6.png]
